# 

**Published:** 1897-04

**Authors:** 


### CONTENTS.

COMMUNICATIONS.

Abrasions of the Teeth........ 157
Nodule on Apex of Root........ 168
Report of Committee on Nomenclature........ 169
Distribution of Exostosis on the Teeth........ 174
Manifestations of Abrasion........ 176
Section II.—Report on Dental Education........ 178

SELECTIONS.

How to Breathe........ 183
The Spitting Nuisance........ 185
The Sickness Rate in Germany........ 187
Some Reminders in Treatment and Diagnosis........ 188
Congenital Teeth........ 189
Absolute Alcohol as a Disinfectant for Surgical Instruments........ 191
Eucain........ 192
Euchinin—A New Preparation of Quinin........ 193
The Antiseptic Properties of Saliva........ 194
How to Write a Medical Paper........ 195
Multum in Parvo........ 195
Life and Death in the Emotions........ 196
Roentgen Rays are Quite Complex........ 196
The Art of not Hearing........ 197
Surgical Items........ 197
Phlegmon of the Hand........ 198
Want a State Prosecuting Agent........ 198
Is Consumption Disappearing........ 199
United States Supreme Court........ 199
Cocaine........ 200

EDITORIAL.

What of Cataphoresis?—Is it Practicable?—Is it Desirable?........ 201
Commencements—Dental Colleges........ 202
The Odontographic Society of Cincinnati........ 204
Bibliographical........ 204

BIOGRAPHICAL.

Dr. S. B. Browu Dead—Was the Oldest Practicing Dentist in Fort Wayne........ 206

